# Developing a lncRNA Signature to Predict the Radiotherapy Response of Lower-Grade Gliomas Using Co-expression and ceRNA Network Analysis

**DOI:** 10.3389/fonc.2021.622880

**Published:** 2021-03-09

**Authors:** Zhongyang Li, Shang Cai, Huijun Li, Jincheng Gu, Ye Tian, Jianping Cao, Dong Yu, Zaixiang Tang

**Affiliations:** ^1^ School of Radiation Medicine and Protection, Soochow University Medical College (SUMC), Suzhou, China; ^2^ Department of Radiotherapy and Oncology, The Second Affiliated Hospital of Soochow University, Suzhou, China; ^3^ Institute of Radiotherapy and Oncology, Soochow University, Suzhou, China; ^4^ Department of Biostatistics, School of Public Health, Medical College of Soochow University, Suzhou, China; ^5^ Jiangsu Provincial Key Laboratory of Geriatrics Prevention and Translational Medicine, School of Public Health, Soochow University Medical College, Suzhou, China; ^6^ School of Radiation Medicine and Protection and Collaborative Innovation Center of Radiation Medicine of Jiangsu Higher Education Institutions, Soochow University, Suzhou, China

**Keywords:** The Cancer Genome Atlas, low-grade glioma, bioinformatics, long non-coding RNA, radiosensitivity

## Abstract

**Background:**

Lower-grade glioma (LGG) is a type of central nervous system tumor that includes WHO grade II and grade III gliomas. Despite developments in medical science and technology and the availability of several treatment options, the management of LGG warrants further research. Surgical treatment for LGG treatment poses a challenge owing to its often inaccessible locations in the brain. Although radiation therapy (RT) is the most important approach in this condition and offers more advantages compared to surgery and chemotherapy, it is associated with certain limitations. Responses can vary from individual to individual based on genetic differences. The relationship between non-coding RNA and the response to radiation therapy, especially at the molecular level, is still undefined.

**Methods:**

In this study, using The Cancer Genome Atlas dataset and bioinformatics, the gene co-expression network that is involved in the response to radiation therapy in lower-grade gliomas was determined, and the ceRNA network of radiotherapy response was constructed based on three databases of RNA interaction. Next, survival analysis was performed for hub genes in the co-expression network, and the high-efficiency biomarkers that could predict the prognosis of patients with LGG undergoing radiotherapy was identified.

**Results:**

We found that some modules in the co-expression network were related to the radiotherapy responses in patients with LGG. Based on the genes in those modules and the three databases, we constructed a ceRNA network for the regulation of radiotherapy responses in LGG. We identified the hub genes and found that the long non-coding RNA, DRAIC, is a potential molecular biomarker to predict the prognosis of radiotherapy in LGG.

## Introduction

Gliomas are the most prevalent malignant primary brain tumors accounting for 81% of all malignant brain tumors (1). The World Health Organization (WHO) has classified gliomas into four grades; WHO grade II and III gliomas are not as malignant as WHO grade IV glioblastoma (GBM). Therefore, WHO grade II and grade III gliomas are defined as lower-grade gliomas (LGG) by The Cancer Genome Atlas (TCGA). Lower-grade gliomas include astrocytomas, oligodendrogliomas, and oligoastrocytomas (2).

Standard treatment of LGG includes surgery, chemotherapy, and radiation therapy. Because lower-grade gliomas occur primarily in the functional areas of the brain and tend to grow aggressively with diffuse infiltration, the suitability of surgery is often controversial. Chemotherapy with temozolomide has some limitations (such as hematological toxicity and myelosuppression (3, 4)). Radiation therapy has significant advantages in the treatment of LGG. Almost all patients with LGG receive radiation therapy during their treatment (5).

Although radiotherapy is associated with several advantages in the treatment of LGG, there exists the problem of heterogeneity in the efficacy of radiotherapy. Patients who receive radiation therapy show varying responses; some show better short-term responses and overall survival compared to others (6). Moreover, side effects such as cognitive abnormality and seizure due to the brain damage caused by ionizing radiation have been observed in some patients (7, 8). With progress in precision medicine, the study of biomarkers for use in radiation therapy and the molecular mechanisms regulating the sensitivity of radiation therapy have gradually become the focus of research in radiation oncology in recent times.

Long non-coding RNAs (lncRNA) belong to a class of non-coding RNA with a length of not more than 200 nucleotides and usually lack coding potential. Several studies have confirmed that lncRNA expression is associated with tumor initiation, progression, and treatment (9–13). Some lncRNAs have also been implicated in the regulation of tumor radiosensitivity. For example, lncRNA CYTOR sponges miR-195 to regulate the radiosensitivity of non-small cell lung cancer (NSCLC) (14). And lncRNA GAS5 can interact with miR-21 and enhance radiosensitivity in NSCLC (15) whereas lncRNA ANRIL enhances the radiosensitivity of nasopharyngeal carcinoma *via* miR-125a (16). Collectively, these studies reveal that lncRNAs can modulate tumor radiosensitivity by functioning as competitive endogenous RNA (ceRNA).

The mechanism of ceRNA is a hypothesis that some RNAs, such as lncRNA, act as a molecular sponge and compete with mRNA for binding to miRNA *via* the miRNA response element (MRE) (17). Although increasing research on ceRNA reveals its role in the progression of many diseases and the treatment responses (18, 19), few studies pertaining to the radiosensitivity of LGG currently focus on the regulatory function of their non-coding RNAs or the mechanism of ceRNAs. Therefore, additional systematic studies on the mechanisms of the regulation of radiosensitivity in LGG are needed.

In this study, we used weighted correlation network analysis (WGCNA) to screen the most relevant modules in the co-expression network and construct a ceRNA network. WGCNA is a systems biology method used to detect the co-expression of gene modules (20, 21) and genes in the same module having a similar expression mode. This technique has been widely used in biological research. Our study provides clues to determine the mechanism of post-transcriptional regulation in LGG radiosensitivity using transcriptome level data. Through analysis of the expression level of hub genes in the co-expression network, we found a lncRNA as a potential biomarker that can be used to predict the prognosis of patients with LGG undergoing radiotherapy.

## Data Sources and Methods

### Data Sources

Gene expression data and clinical follow-up data from patients with LGG were downloaded from The Cancer Genome Atlas (TCGA). TCGA is the world’s largest oncogene database, providing a large number of gene expression data, mutation data, epigenetic data, clinical data, and survival data of different tumors.

The expression levels in the RNA-seq data are normalized by TCGA. We directly used the data standardized by Fragments Per Kilobase per Million (FPKM) provided by TCGA as the expression level of the gene.

We categorized patients into radiosensitive and radioresistant groups based on the short-term response of their primary tumor to radiotherapy. Patients who showed complete remission after radiotherapy were considered radiosensitive whereas those exhibiting disease progression after radiotherapy were considered resistant to radiotherapy. For survival analysis, our inclusion criteria for patients were follow-up survival time greater than 30 days and those who had received radiation therapy.

The lncRNA and mRNA expression data were extracted from RNA-seq expression data of TCGA-LGG according to the GENCODE (https://www.gencodegenes.org/) annotations database V34.

To validate our findings of the biomarkers related to TCGA-LGG radiosensitivity, we performed overall survival validation using two independent datasets of Chinese Glioma Genome Atlas (CGGA, http://www.cgga.org.cn). The expression of the two CGGA datasets was sequence matched using STAR (22) and transcripts were quantified using RSEM (23). The two CGGA datasets included 325 (24, 25) and 693 patients with glioma (26, 27), respectively.

In both CGGA datasets, patients were screened based on criteria, such as glioma grade WHO II and III, whether or not they received radiation therapy, and survival follow-up longer than 30 days.

The clinical data of patients from TCGA and CGGA are uploaded as supplementary material.

### WGCNA Co-expression Analysis

Co-expression network analysis was conducted using the “WGCNA” package in R 4.0 software. Genes with a low amplitude of change and low expression are generally not considered to play a critical biological role in the regulation of organismal function and in improving the computational efficiency of WGCNA. The filter standard of miRNA is a median absolute deviation (MAD) higher than 0.01. MAD is a robust statistic used to describe the dissociation between samples. For lncRNA and mRNA, the top 5000 lncRNA and mRNA with high MAD were selected. Hierarchical clustering analysis was conducted to remove the outliers.

We performed a co-expression network analysis on lncRNA, mRNA, and miRNA expression levels. First, the value of the powers (beta) was estimated using the “pickSoftThreshold” function in the WGCNA package. The R-squared criterion was set to 0.9. Pearson correlation coefficients were calculated using the expression data to generate a correlation matrix, which was converted to a weighted adjacency matrix based on the power. Lastly, a topological overlap matrix (TOM) was generated to describe the connection between genes. Genes with high co-expression were then grouped into same modules based on the TOM. The merge cut-off threshold was set to 0.2, which meant that modules with a similarity higher than 0.8 were merged into one module.

### Module-Radiosensitivity Relationship

Principal component analysis (PCA) of the modules in the co-expression network of lncRNA, mRNA, and miRNA was performed. The first principal component (Eigengene) represented the gene expression level within the module and was used for Pearson correlation analysis for radiosensitivity. The modules with the strongest correlation and p-value < 0.05 were considered to play a key role in radiosensitivity.

### ceRNA Network Construction and Visualization

We predicted these genes using three RNA interaction databases, including lncBase (http://carolina.imis.athena-innovation.gr/diana_tools/web/index.php?r=lncbasev2%2Findex-predicted), miRDB (http://mirdb.org/), and mirTarbase (http://mirtarbase.cuhk.edu.cn/php/index.php). The lncBase was used to predict the interaction of lncRNA with miRNA, whereas miRDB and mirTarbase were used to predict the interaction of miRNA with mRNA. The threshold for the miTGscore in the lncBase was set to 0.9. Interaction pairs with an miTGscore above 0.9 were considered reliable and were included in the construction of the ceRNA network. The target mRNAs of miRNAs were predicted using miRDB and mirTarbase, and the sum aggregate of these two databases was considered as the target of miRNA. The R package “ggalluvial” (28) was used for the visualization of the ceRNA network.

### Gene Ontology and Pathway Enrichment Analysis

Gene ontology (GO) and Kyoto Encyclopedia of Genes and Genomes (KEGG) enrichment analysis of target genes in the ceRNA network were implemented using the R package “clusterprofiler” (29). GO enrichment analysis included three ontologies, namely, biological process (BP), molecular function (MF), and cellular component (CC). The p-value of GO and KEGG enrichment analysis was adjusted using the Benjamini-Hochberg method. The R package “GOplot” (30) was used to visualize the GO enrichment data.

### Selection of Hub Genes

To further screen biomarkers, RNA within the three modules were identified as hub genes. Hub genes are considered to be genes with high connectivity within the module that play a key pivotal role in regulation and are, therefore, more meaningful as biomarkers. Gene significance (GS) and module membership (MM) were calculated for each gene. The selection criteria for hub genes were set to GS > 0.2 and MM > 0.8.

### Survival Analysis

To identify the relationship between the expression level of these hub genes and patient prognosis after radiotherapy, all patients who had received radiotherapy and had valid survival data were selected for survival analysis. Patients were divided into high and low groups based on the expression level of each gene. Kaplan-Meier curves and log-rank test were used for survival analysis to calculate the effect of the expression of each gene on the prognosis of patients with LGG who had received radiotherapy. Survival analysis and visualization were performed using the “survival” (31) and “survminer” R package. The p-value was adjusted using the false discovery rate.

## Results

### Processing of Data

This study included 49 patients with LGG (**Table 1**), among whom 30 had gliomas that showed complete response after radiotherapy and 19 showed radiographic progressive disease. The RNA-seq expression data of all patients were available, but because the miRNA-seq data of one of the patients in the complete response group was missing, only 48 patients were included for the miRNA co-expression network analysis.

**Table 1:** Patient characteristics (n=49).

	Progressive disease group	Complete Response group
Total	19 (100%)	30 (100%)
Age		
>40	11 (57.90%)	20 (66.67%)
≦40	8 (42.10%)	10 (33.33%)
Grade		
II	7 (36.84%)	6 (25.00%)
III	12 (63.16%)	24 (75.00%)
Gender		
Male	11 (57.90%)	12 (40.00%)
Female	8 (42.10%)	18 (60.00%)
IDH1		
Mutation	5 (26.32%)	6 (20.00%)
Wild	3 (15.79%)	4 (13.33%)
NA	11 (57.89%)	20 (66.67%)
RT dose		
≥5400cGy	17 (89.47%)	29 (96.67%)
<5400cGy	2 (10.53%)	1 (3.33%)

A total of 19,600 mRNA and 14,085 lncRNA were identified using GENCODE annotation database v34. The MAD of genes were calculated. There were a total of 2142 miRNAs in the miRNA expression data, of which 792 had MADs greater than 0.01. The top 5000 lncRNA and mRNA with larger MAD were extracted for further analyses.

### WGCNA Analysis

**Figure 1:**
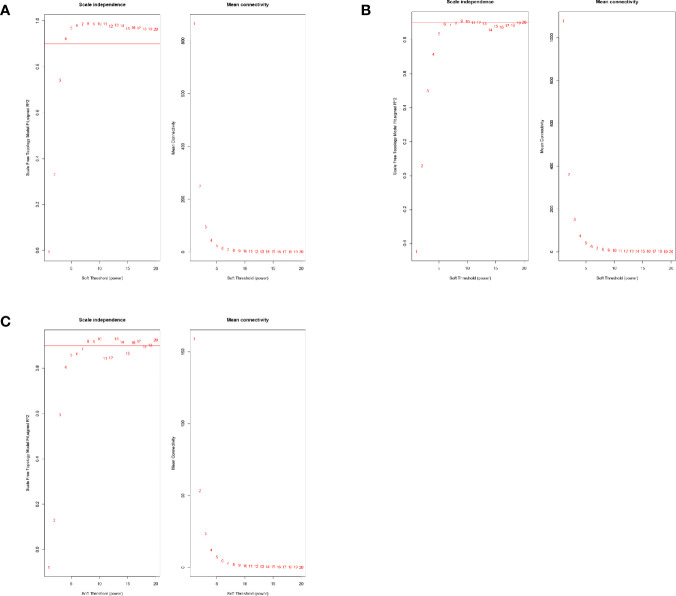
**(A)** The power value selection of lncRNA co-expression networks. **(B)** The power value selection of mRNA co-expression networks. **(C)** The power value selection of miRNA co-expression networks.

The mRNA expression data of one of the patients in the complete response group was identified as an outlier in hierarchical clustering analysis and was removed. Beta value is key to build a high-efficiency co-expression network to find the most relevant module in WGCNA analysis. The power value was calculated using the function “pickSoftThreshold.” The minimum R-squared value was set to 0.9 (**Figure 1**). The beta value of lncRNA for the construction of the co-expression network was set to 4, whereas it was set to 9 and 8 for mRNA and miRNA, respectively.

**Figure 2:**
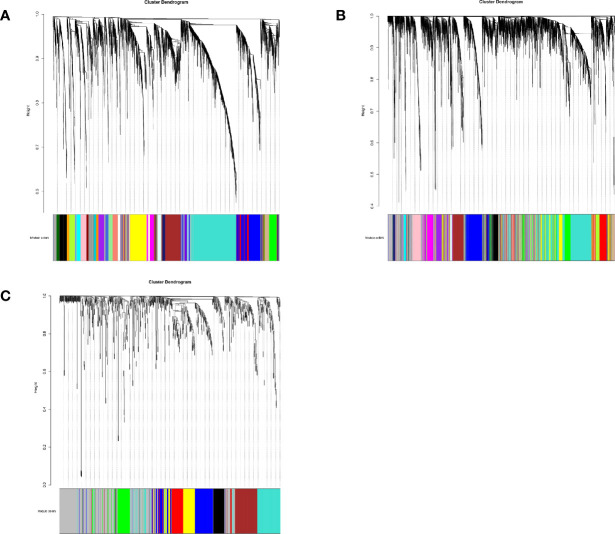
**(A)** The cluster dendrogram of lncRNA co-expression network. **(B)** The cluster dendrogram of mRNA co-expression network. **(C)** The cluster dendrogram of miRNA co-expression network.

**Figure 3:**
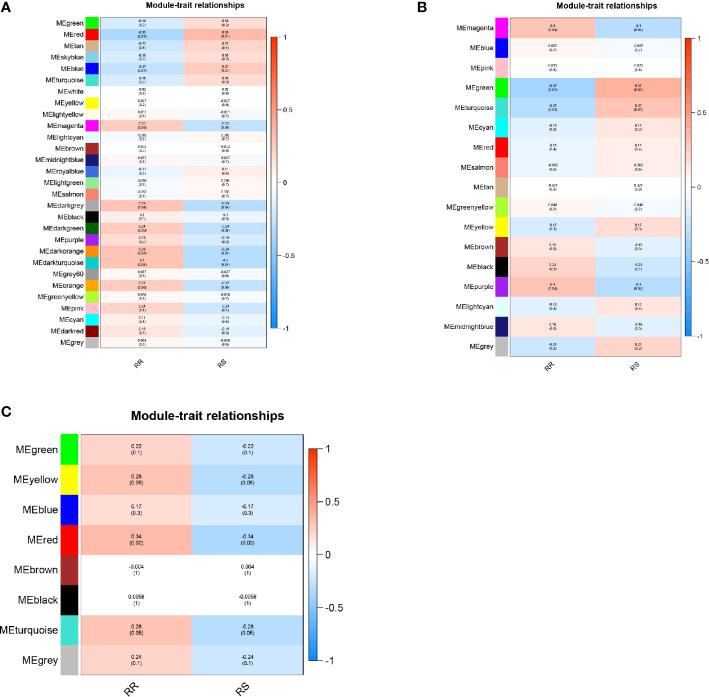
**(A)** Module‐trait relationship of lncRNA co-expression network. **(B)** Module‐trait relationship of mRNA co-expression network. **(C)** Module‐trait relationship of miRNA co-expression network.

A total of 29 modules were identified from the lncRNA co-expression network. Seventeen mRNA modules from the mRNA co-expression network and 8 miRNA modules from miRNA co-expression network are shown in **Figure 2**. In the module-trait correlation analysis, the lncRNA module, MEred, the mRNA module, MEgreen, and the miRNA module, MEred, are the modules that are most correlated to the radiotherapy response of patients (**Figure 3**). The genes in these three modules are highly related to radiotherapy response in LGG.

### ceRNA Network Analysis

**Figure 4:**
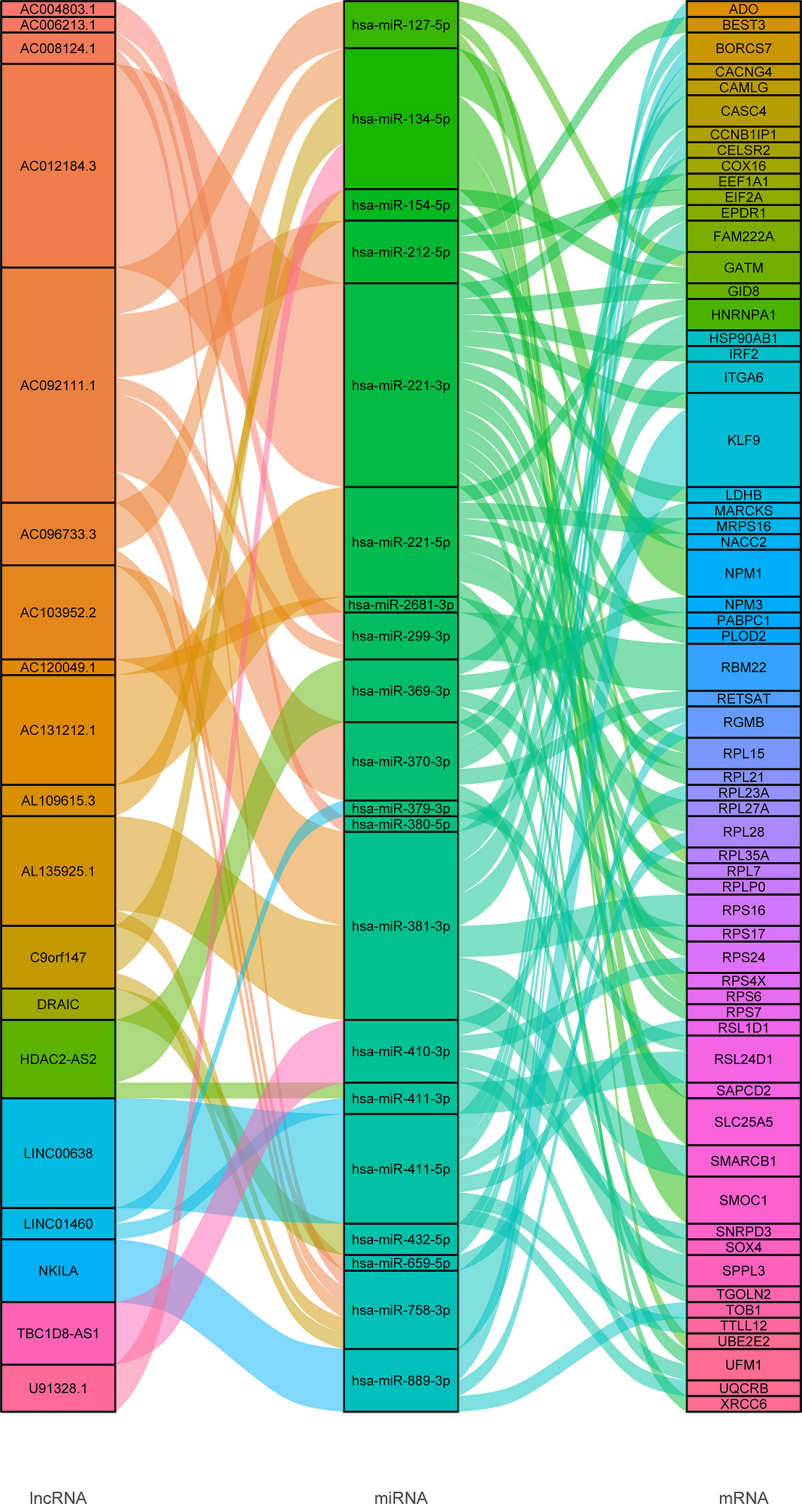
Sankey diagram of ceRNA network.

Using the Lncbase database, 3142 lncRNA-miRNA interaction pairs were predicted by lncRNA in MEred. Among those, 32 lncRNA-miRNA interaction pairs were related to 21miRNA in module MEgreen. MiRDB and mirTarBase were used to predict the target mRNAs of the 21miRNAs. There were 21 and 53 interaction pairs between miRNA and mRNA found in the miRDB and miRTarBase, respectively. The miRNA-mRNA predictions were combined and 19 lncRNAs, 20 miRNAs, and 61 mRNAs were included in the ceRNA network (**Figure 4**).

### GO and KEGG Pathway Enrichment Analysis

**Figure 5:**
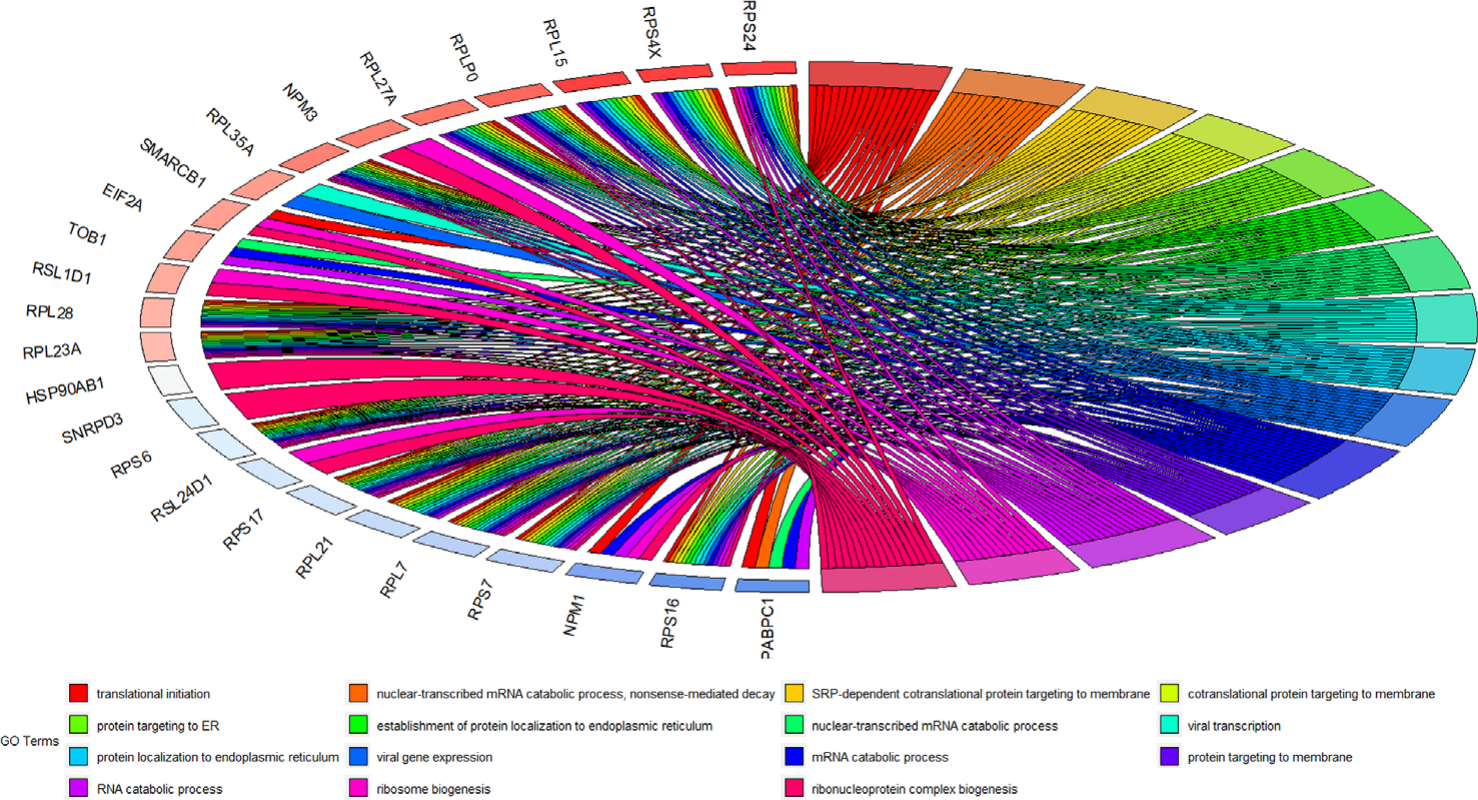
GO terms of 61 target mRNAs in ceRNA network.

**Figure 6:**
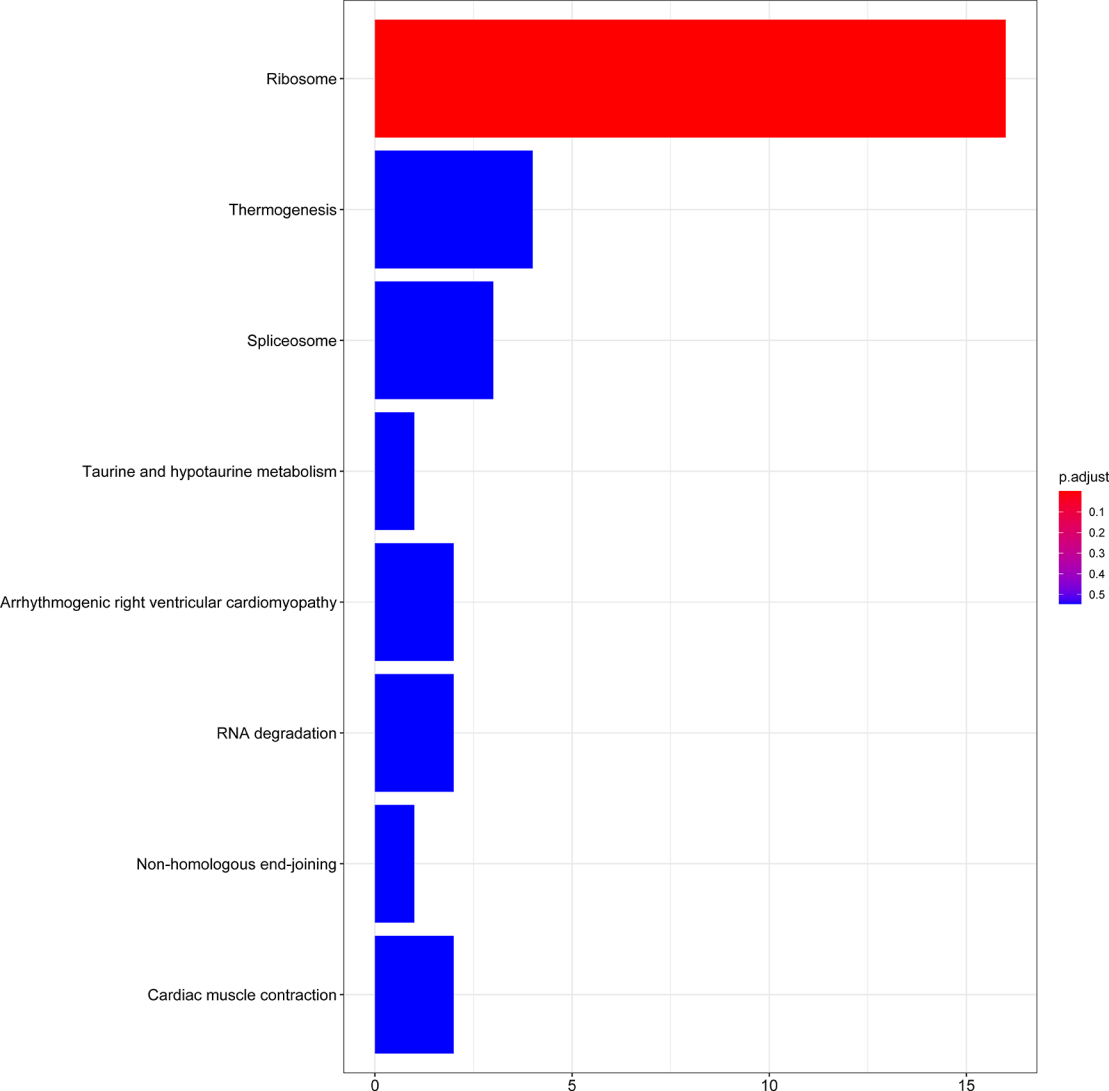
KEGG enrichment analysis of 61 target mRNAs.

A total of 56 GO terms were identified from 61 target mRNAs. The target mRNAs in ceRNA were primary associated with GO terms such as translational inhibition, negative regulation of ubiquitin-dependent protein catabolic process, and positive regulation of translation (**Figure 5**). The most significant KEGG pathway that the target mRNA was associated with was the ribosome pathway (**Figure 6**).

### Hub Gene Selection and Survival Analysis

**Figure 7:**
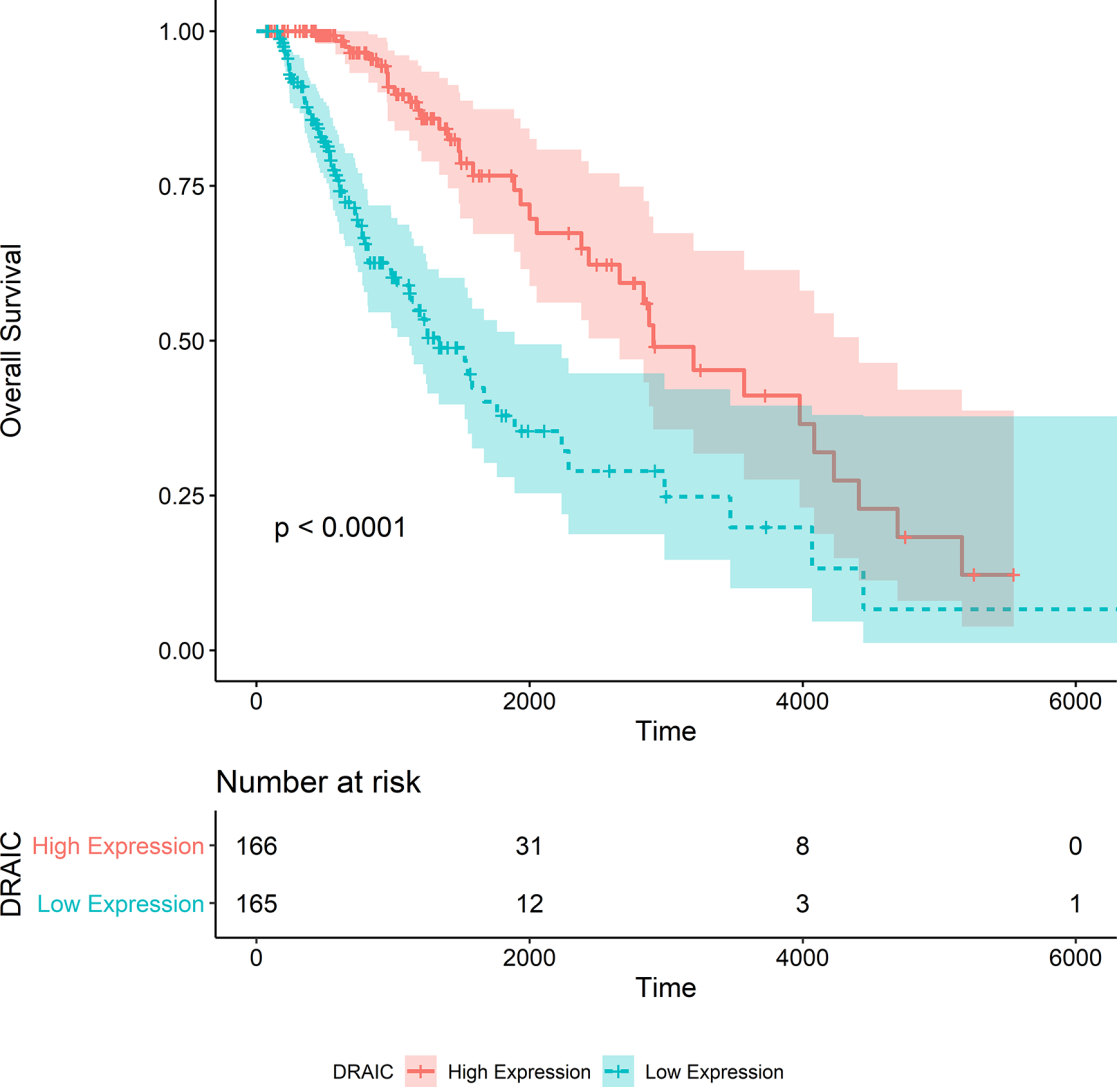
Kaplan-Merier survival curve of overall survival in the TCGA-LGG dataset.

After the calculation of GS and MM, 13 lncRNAs, 28 miRNAs, and 74 mRNAs were selected as hub genes. Results from the survival analysis (**Table 2**) indicated that DRAIC was the most significant lncRNA affecting the overall survival (OS) of patients who had received radiotherapy. The group with high lncRNA DRAIC expression showed a significantly better overall survival than that with low lncRNA DRAIC expression (p < 0.0001) (**Figure 7**).

**Table 2:** Survival analysis results of hub lncRNAs.

Ensembl ID	Gene Symbol	p-value
ENSG00000203497	PDCD4-AS1	0.023584
ENSG00000229980	TOB1-AS1	0.033365
ENSG00000239415	AP001469.3	0.000122
ENSG00000245750	DRAIC	1.24E-07
ENSG00000253669	GASAL1	0.019953
ENSG00000260830	AL135744.1	0.033365
ENSG00000261777	AC012184.3	0.404931
ENSG00000262362	AC004233.1	0.010548
ENSG00000270403	AP001554.1	0.019953
ENSG00000272079	AC004233.2	0.019953
ENSG00000274367	AC004233.3	0.050642
ENSG00000277182	AC006449.5	0.010548
ENSG00000278012	AL031658.2	0.000122

**Figure 8:**
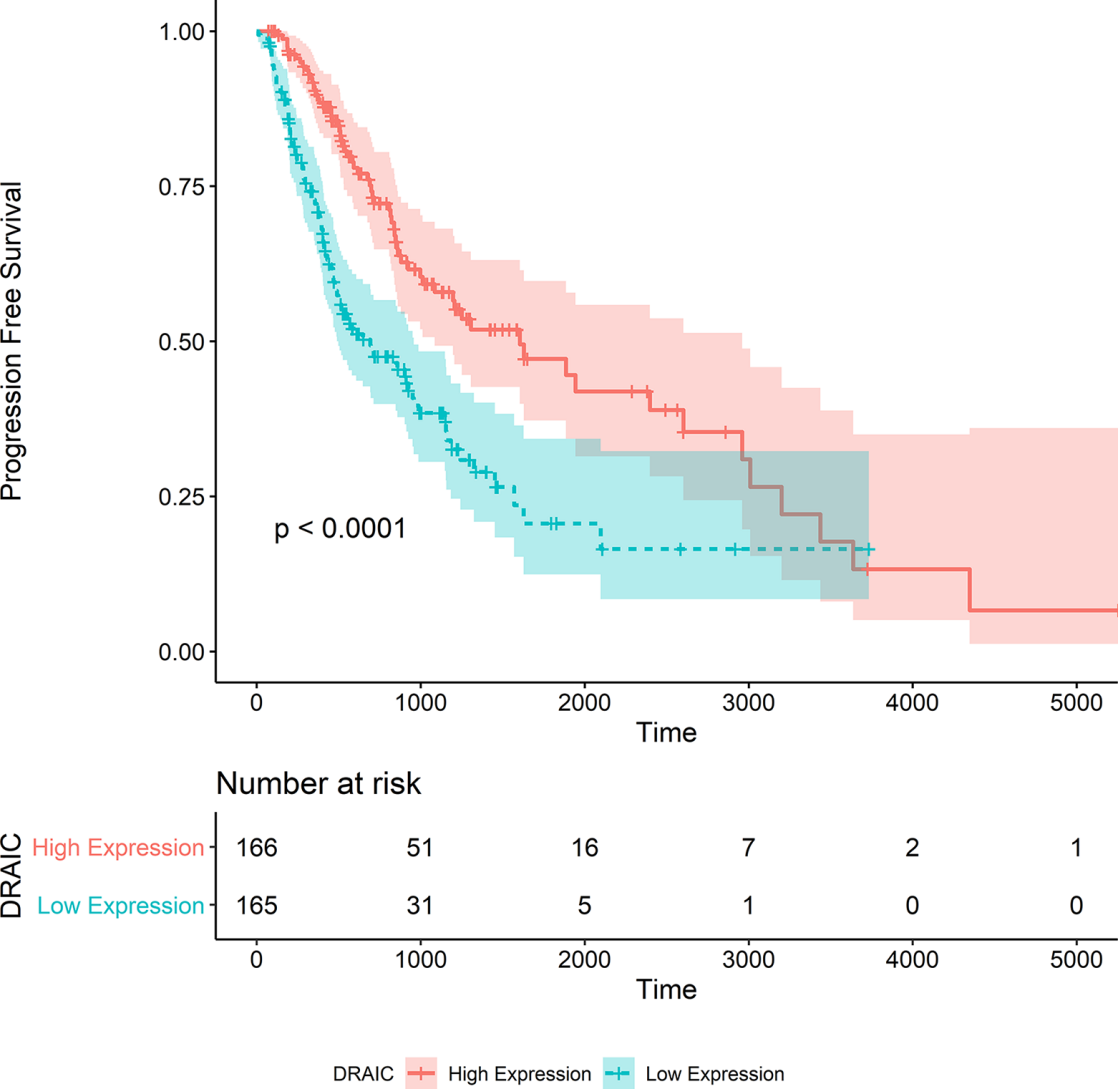
Kaplan‐Meier survival curve of progression free survival in the TCGA-LGG dataset.

We also noticed that the group with high lncRNA DRAIC expression level exhibited better progression-free survival than that with the low expression level of lncRNA DRAIC (p < 0.0001) (**Figure 8**).

Two CGGA datasets were used as independent datasets to validate the relationship between the expression level of lncRNA DRAIC and the OS of patients with LGG. From the CGGA325 dataset, we extracted the data of 137 patients with WHO grade II and III tumors with survival follow-up greater than 30 days who had received radiation therapy. We also extracted the data of 308 patients from the CGGA693 dataset based on similar criteria.

**Figure 9:**
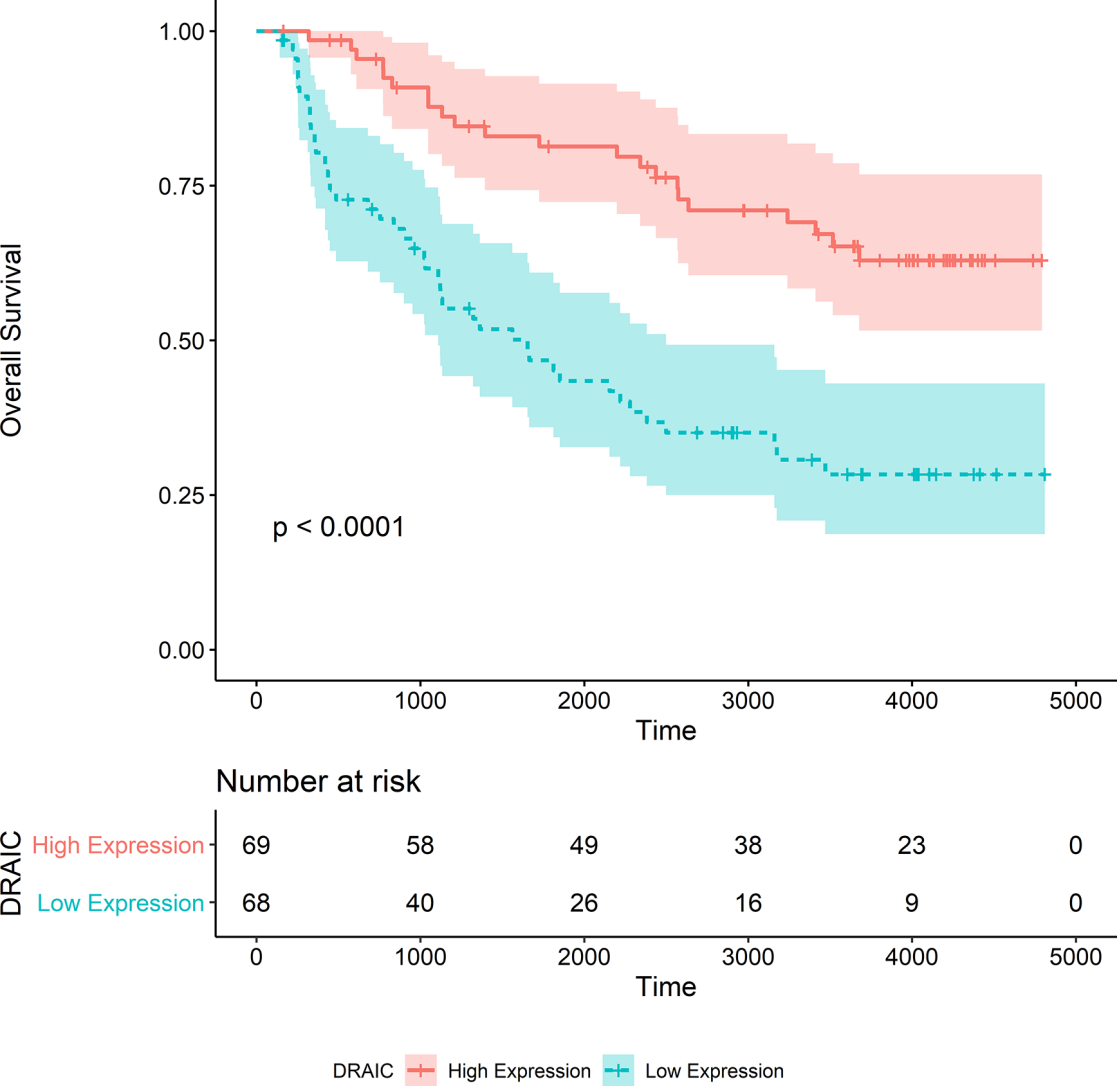
Kaplan-Merier survival curve of overall survival in the CGGA325 dataset.

**Figure 10:**
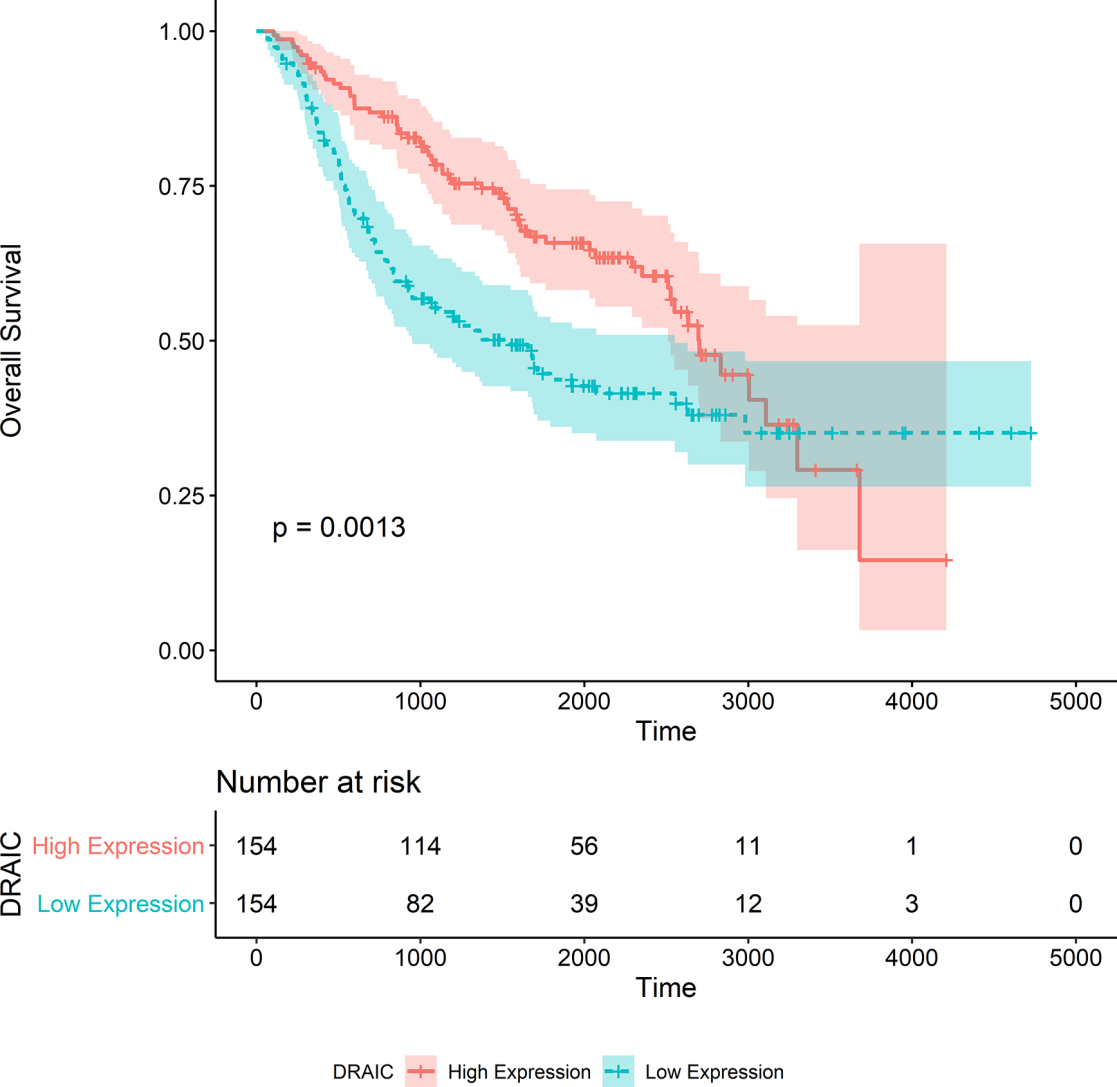
Kaplan-Merier survival curve of overall survival in the CGGA693 dataset.

The OS data of patients with high DRAIC expression obtained from the CGGA325 dataset was significantly better than those of patients in the low expression group (p<0.0001) (**Figure 9**). Although the long-term survival of patients was not significantly better in the DRAIC high expression group, the OS and five-year survival were significantly better than that in the DRAIC low expression group in the CGGA693 dataset (p=0.0013) (**Figure 10**).

Chi-square test was used to evaluate the relationship between DRAIC expression and levels of the traditional biomarkers in the CGGA datasets. We found that the expression level of lncRNA DRAIC was highly correlated with IDH mutation and 1p/19q codeletion. In both CGGA datasets, the DRAIC high expression group had more 1p/19q codeletion and IDH1 mutations compared to those in the low expression group. However, lncRNA DRAIC expression was not related to MGMT methylation (**Tables 3** and **4**).

**Table 3:** Relationship between lncRNA DRAIC expression and 1p/19q, IDH mutation, and MGMT methylation in CGGA325 dataset.

CGGA325 dataset	High DRAIC expression group (n=69)	Low DRAIC expression group (n=68)	p-value
1p/19q			<0.0001
Codel	40	10	
Non-codel	28	58	
IDH			<0.0001
Mutant	65	37	
Wildtype	4	31	
MGMT			0.7483
Methylated	37	32	
Unmethylated	27	28	

**Table 4:** Relationship between lncRNA DRAIC expression and 1p/19q, IDH mutation, and MGMT methylation in CGGA693 dataset.

CGGA693 dataset	High DRAIC expression group (n=154)	Low DRAIC expression Group (n=154)	p-value
1p/19q			<0.0001
Codel	67	23	
Non-codel	68	122	
IDH			<0.0001
Mutant	132	86	
Wildtype	13	50	
MGMT			0.1311
Methylated	79	69	
Unmethylated	40	54	

## Discussion

The response of patients who receive radiotherapy for tumors varies widely. Radiotherapy induces several effects including double-strand breaks (DSB) in the DNA, DNA damage repair, and the generation of oxygen radicals by the ionizing radiation (32, 33). The sensitivity of individuals to radiotherapy varies widely and depends on several factors. Each patient responds differently and the nature of the response to radiotherapy is highly dependent on the genetic makeup (34, 35). Radiotherapy sensitivity has been one of the most important topics of research in radiation oncology for a long time. However, few studies have focused on the regulation of radiosensitivity at the post-transcriptional level. Molecular biomarkers, such as IDH1 and IDH2 mutation (36–39) and 1p19q codeletion (40, 41), have been used to predict the prognoses of patients with LGG. The molecular mechanism involved in the regulation of radiation response in patients with LGGs is still undefined and, to date, there is no effective or reliable biomarker that can be used to determine the prognosis of patients undergoing radiotherapy.

In this study, for the first time, we systematically investigated the mechanism of ceRNA regulation in the radiosensitivity of LGG based on RNA-seq data and database predictions. Consequently, a lncRNA was identified as a biomarker that could be effective in predicting the prognosis of patients after radiotherapy.

After obtaining data from TCGA-LGG, we categorized patients into different groups based on their short-term response of their primary tumor to radiotherapy. Although the TCGA-LGG project did not provide details of surgical resection, we believe that for low-grade gliomas, even if maximum resection is performed (e.g., gross total resection), some microscopic lesions may still be present. These residual microscopic lesions may still have the potential for local recurrence and distal metastasis. This is one of the reasons why lower-grade gliomas are treated using radiotherapy after surgery. However, patients may still present differently after postoperative radiotherapy, and some patients may develop local recurrence and distant metastases (42). Therefore, TCGA takes into consideration not only the imaging performance of the lesion before and after radiotherapy but also new tumor events when assessing the response to radiotherapy. Complete response is defined as the disappearance of all target lesions after receiving radiotherapy without the formation of new lesions for at least 4 weeks. Also, by reviewing the survival and follow-up data of patients in the CR group, we found that the majority of patients in the CR group had no new tumor events during their long-term follow-up. Therefore, we believe that TCGA is accurate in assessing the recovery of patients and the efficacy of the treatment modality, and our practice of using the short-term response to radiotherapy in TCGA to group patients is reasonable.

Normally, the RNA-seq studies involve gene analyses to identify genes related to the trait. All gene expression levels are analyzed using differential gene expression analysis and the differentially expressed genes (DEGs) are selected based on a foldchange threshold. However, the foldchange threshold is not the ideal choice in biology research as there is no significant difference in the function of a gene with expression levels a little higher or lower than the foldchange threshold. Therefore, in this study, we chose WGCNA analysis to discover the important genes that are involved in the radiosensitivity in LGG. The WGCNA algorithm avoids the problem of threshold by using a soft threshold. In WGCNA analysis, the correlation coefficient of all genes is taken as the power of n, the coefficient distribution conforms to the scale-free network, and the genes are classified into different modules based on the mode of expression. Genes in the same module exhibit highly similar expression. The distribution pattern of nodes in the scale-free network corresponds to the mode of action of genes and has a biological significance, which is the advantage of using the WGCNA algorithm.

Using WGCNA analysis, we observed that the most relevant modules of lncRNA and mRNA were positively correlated with radiosensitivity and the most relevant module of miRNA was negatively correlated with radiosensitivity. These findings were consistent with the competitive binding mechanism of ceRNA. In the gene function enrichment analysis, we noticed that most of the functions of the target mRNAs in the ceRNA network were highly concentrated in the ribosomal pathway. Currently, the role of ribosomes in the response of tumor cells to ionizing radiation has not been elucidated in the field of gene research pertaining to radiosensitivity.

We noticed that lncRNA DRAIC had the most significant effect in predicting the prognosis of patients after receiving radiotherapy; lncRNA DRAIC has been shown to inhibit the progression of prostate cancer by interacting with IκB kinase (IKK) and inhibiting NF-κB activity (43). Activation of NF-κB is associated with the radiosensitivity of gliomas (44–46). DRAIC might be the key lncRNA involved in the radiosensitivity regulation of LGG. Studies report that DRAIC can be a biomarker to predict prognosis in many malignancies (47). However, there is no direct evidence to confirm the involvement of lncRNA DRAIC in the regulation of radiosensitivity in LGG; therefore, further studies are warranted.

To further strengthen the conclusions based on the data obtained from TCGA dataset, we performed independent validation of the OS in patients who underwent radiotherapy. To this effect, we used two CGGA datasets to validate DRAIC as a biomarker of the response to radiotherapy. The conclusions obtained based on both CGGA datasets were similar to those derived from TCGA, which indicated that patients in the high DRAIC expression group would achieve better OS after radiation therapy compared to those in the low-expression group. Furthermore, we noticed that in the CGGA datasets, IDH mutation and 1p/19q codeletion status were highly correlated with lncRNA DRAIC expression. Previous studies have shown that IDH mutation and 1p/19q codeletion are related to the radiosensitivity of gliomas (48–50). IDH mutation and 1p/19q codeletion increase the radiosensitivity of gliomas. These results are in agreement with our findings that lncRNA DRAIC can be used as a potentially suitable biomarker to determine radiosensitivity in patients.

Our study has some limitations. Although the number of patients who were included in this study based on their specific response to radiotherapy is justified and adequate for WGCNA analysis, additional samples may help increase the confidence levels of our findings. *In vivo* and *in vitro* studies (such as knockdown/knockout of DRAIC and molecular functional tests) can help further corroborate the conclusions of our study. This will be the focus of our subsequent study.

## Data Availability Statement

Publicly available datasets were analyzed in this study. These data can be found here: https://portal.gdc.cancer.gov/repository, http://www.cgga.org.cn/download.jsp.

## Author Contributions

Study conception and design: ZL, ZT, and DY. Real data and analysis: ZL, SC, and JG. Drafting of the manuscript: ZL, SC, JG, YT, and JC. All authors contributed to the article and approved the submitted version.

## Funding

This work was supported in part by the National Natural Science Foundation of China (81773541), funded from the Priority Academic Program Development of Jiangsu Higher Education Institutions at Soochow University, the State Key Laboratory of Radiation Medicine and Protection (GZK1201919) to ZT; a project by the Second Affiliated Hospital of Soochow University (XKTJ-RC202007), Scientific Research Program for Young Talents of China National Nuclear Corporation (51003), Suzhou Science and Education Project (KJXW2017010), The Natural Science Foundation of Jiangsu Province (BK20180195), the National Natural Science Foundation of China (81902715) to SC; National Natural Science Foundation of China (U1967220 and 81872552) to JC; Jiangsu Provincial Key Project in Research and Development of Advanced Clinical Technique (BL2018657) to YT; and the Natural Science Foundation of Jiangsu Province (BK2016), National Natural Science Foundation of China (11475125), and The Starting Research Fund from the Soochow University (Q412600711) to DY. The funding body did not play any roles in the design of the study and collection, analysis, and interpretation of data and in writing the manuscript.

## Conflict of Interest

The authors declare that the research was conducted in the absence of any commercial or financial relationships that could be construed as a potential conflict of interest.
